# Posttraumatic Stress Disorder: Overview of Evidence-Based Assessment and Treatment

**DOI:** 10.3390/jcm5110105

**Published:** 2016-11-22

**Authors:** Cynthia L. Lancaster, Jenni B. Teeters, Daniel F. Gros, Sudie E. Back

**Affiliations:** 1 Ralph H. Johnson Veterans Affairs Medical Center, 109 Bee Street, Charleston, SC 29401, USA; lancascy@musc.edu (C.L.L.); teeters@musc.edu (J.B.T.); grosd@musc.edu (D.F.G.); 2 Department of Psychiatry and Behavioral Sciences, Medical University of South Carolina, 5 Charleston Center Drive, Suite 151, Charleston, SC 29401, USA

**Keywords:** posttraumatic stress disorder, evidence based, empirically supported, assessment, psychotherapy, pharmacotherapy, prolonged exposure, cognitive processing therapy, eye movement desensitization and reprocessing, selective serotonin reuptake inhibitors

## Abstract

Posttraumatic stress disorder (PTSD) is a chronic psychological disorder that can develop after exposure to a traumatic event. This review summarizes the literature on the epidemiology, assessment, and treatment of PTSD. We provide a review of the characteristics of PTSD along with associated risk factors, and describe brief, evidence-based measures that can be used to screen for PTSD and monitor symptom changes over time. In regard to treatment, we highlight commonly used, evidence-based psychotherapies and pharmacotherapies for PTSD. Among psychotherapeutic approaches, evidence-based approaches include cognitive-behavioral therapies (e.g., Prolonged Exposure and Cognitive Processing Therapy) and Eye Movement Desensitization and Reprocessing. A wide variety of pharmacotherapies have received some level of research support for PTSD symptom alleviation, although selective serotonin reuptake inhibitors have the largest evidence base to date. However, relapse may occur after the discontinuation of pharmacotherapy, whereas PTSD symptoms typically remain stable or continue to improve after completion of evidence-based psychotherapy. After reviewing treatment recommendations, we conclude by describing critical areas for future research.

## 1. Introduction

Posttraumatic stress disorder (PTSD) is among the most common mental health disorders in the United States [1]. PTSD is associated with a chronic course and debilitating symptoms. This manuscript reviews the epidemiology and clinical characteristics of PTSD, current options for screening and treatment, and describes more recent directions in treatment research. 

### 1.1. Epidemiology 

PTSD develops after exposure to a potentially traumatic event. According to the *Diagnostic and Statistical Manual of Mental Disorders* (DSM; [2]), the traumatic event must involve exposure to actual or threatened death, serious injury, or sexual violence. Exposure is defined as directly experiencing or witnessing a traumatic event, or learning that a trauma occurred to a close family member or friend. PTSD can also develop from repeated or extreme exposure to aversive details of traumatic events, such as military photographers whose job it is to photograph the details of wartime atrocities, first responders who are charged with collecting human remains, and police officers who are repeatedly exposed to details of child abuse. The fifth edition of the diagnostic manual explicitly excludes exposure to traumas via television, movies, pictures, or electronic mediums, possibly due to concerns that the definition of trauma was enlarging to a construct too broad to be useful [2]. Even so, nearly 90% of the general population endorses experiencing one or more traumatic events (with the modal number of trauma exposures being three), such as sexual or physical assault, combat, motor vehicle accidents, and natural disasters [3]. 

Although most individuals experience a traumatic event during their lifetime, the majority of trauma-exposed individuals do not develop PTSD. The lifetime prevalence of PTSD is estimated at 8.3% [3]. During the weeks following a traumatic event, the vast majority of individuals exhibit normative acute reactions, such as intrusive thoughts or dreams about the event, hyper-alertness, irritability, and problems with sleep, memory, and/or concentration [4,5,6,7,8]. For approximately two-thirds of individuals exposed to a traumatic event, these symptoms resolve on their own with time [7,9,10]. PTSD thus is characterized by a failure to follow the normative trajectory of recovery after exposure to a traumatic event. A key to understanding this disorder is therefore investigating predictors of the trajectory of recovery or non-recovery. 

Researchers have identified a dose-response relation between exposure to traumatic events and the subsequent development of PTSD, such that the prevalence of PTSD increases as the number of traumatic events increase [3,11,12]. PTSD is also more likely to occur after more severe types of trauma, such as rape, childhood sexual abuse, or military combat [13]. Furthermore, the population trajectory seems to differ by trauma type. In comparing intentional to non-intentional traumas (as distinguished by whether harm was inflicted deliberately), Santiago and colleagues [10] found that PTSD prevalence increases over time among survivors of intentional trauma, whereas the opposite is true among survivors of non-intentional traumas. 

Higher risk for PTSD has also been associated with numerous pre-trauma variables, including female gender, disadvantaged social, intellectual, and educational status, history of trauma exposure prior to the index event, negative emotional attentional bias, anxiety sensitivity, genetic subtypes implicated in serotonin or cortisol regulation, as well as personal and family history of psychopathology [11,12,14,15,16,17]. PTSD risk factors related to peri-traumatic and post-traumatic variables include perceived life threat during the trauma, more intense negative emotions during or after the trauma (e.g., fear, helplessness, shame, guilt, and horror), dissociation during or after the trauma, lower levels of social support after the trauma, and generally more severe symptoms during the first week following the traumatic event [12,18].

### 1.2. Clinical Characteristics

In addition to a history of trauma exposure, PTSD is characterized by four clusters of symptoms: (1) re-experiencing symptoms (e.g., recurrent intrusive memories, traumatic nightmares, and flashbacks); (2) avoidance symptoms (e.g., avoiding trauma-related thoughts and feelings and/or objects, people, or places associated with the trauma); (3) negative changes in cognitions and mood (e.g., distorted beliefs about oneself or the world, persistent shame or guilt, emotional numbing, feelings of alienation, inability to recall key details of the trauma); and (4) alterations in arousal or reactivity symptoms (e.g., irritability, hypervigilance, reckless behavior, sleep disturbance, difficulty concentrating). In order to qualify for a diagnosis of PTSD, these symptoms must be present for more than one month, lead to significant distress or functional impairment, and must not be due to medications, substance use, or a medical condition. 

## 2. Assessment of PTSD

The thorough assessment of symptoms is an essential component in the effective treatment of PTSD. The primary goals of assessment include the detection of trauma exposure, evaluation of DSM-5 PTSD criteria, and ongoing assessment of symptom severity during treatment [19]. Assessment procedures may involve several steps, ranging from the initial screening typically conducted in non-specialty clinics (e.g., primary care offices) to lengthy diagnostic interviews, and self-report symptom questionnaires. Together, the data gathered through these various methods provides invaluable information that can be used to inform treatment planning and monitor treatment progress. Numerous assessment tools have been developed and investigated for PTSD. The following sections focus on the most common and empirically supported measures relevant to diagnostics, treatment planning, and treatment monitoring for PTSD (see Table 1 for overview). 

### 2.1. Initial Screening

Several brief tools have been developed to screen for exposure to a Criterion A traumatic event, which allows for rapid identification of persons at-risk for PTSD. These screening tools are especially relevant to busy settings that necessitate that a large amount of data be collected in a short period of time, such as primary care clinics [20]. Although there is no gold-standard trauma-exposure screener [19], several options with growing support in the literature exist [21,22]. Questionnaires such as the Trauma Assessment of Adults [21], the Brief Trauma Questionnaire [23], the Life Events Checklist [24], and the Trauma Life Events Questionnaire [22] all have psychometric support for evaluating exposure to potentiality traumatic events. In addition to trauma exposure screeners, abbreviated PTSD symptom screeners are frequently used to determine the need for more in depth clinical interviews. These include the Primary Care PTSD Screen (PC-PTSD; [25]), the Short Form of the PTSD Checklist-Civilian Version [26], the Trauma Screening Questionnaire (TSQ [11]), and the Short Post-Traumatic Stress Disorder Rating Interview (SPRINT [27]).

### 2.2. Diagnosis

After initial screening, more advanced assessment procedures should be conducted to establish clinical diagnosis of PTSD based on the DSM-5 diagnostic criteria. In general, these diagnostic assessments can take up to several hours to complete and require significant training to administer. Although excellent disorder-specific interviews exist for PTSD (Clinician Administered PTSD Scale [28], PTSD Symptom Scale—Interview Version [29]), interviews designed to assess the full spectrum of psychiatric disorders may be better suited for treatment planning due to high rates of psychiatric comorbidity among PTSD patients (e.g., major depressive disorder, substance use disorders, etc.). One of the best examples of this is the Structured Clinical Interview of DSM-5 Disorders (SCID [30]). The SCID has been shown to provide valid and reliable diagnostics for PTSD as well as most other psychiatric disorders. Additional structured clinical interviews include the Anxiety Disorder Interview Schedule for DSM-5 [31], which, unlike the SCID, provides an index to quantify symptom severity, and the Mini International Neuropsychiatric Interview [32], which also provides the DSM diagnoses but takes approximately half the time to administer relative to the SCID. 

### 2.3. Symptom Severity and Treatment Tracking

Once a PTSD diagnosis has been established, symptom frequency and severity are the next essential components to treatment planning and monitoring. A number of measures have been developed for monitoring PTSD symptoms. These measures are generally brief, self-report assessments of the 20 symptoms associated with PTSD. Some of the most widely used measures include the PTSD Checklist for DSM-5 [28] and the Posttraumatic Diagnostic Scale for DSM-5 [29]. These provide quick feedback regarding symptom severity and include cutoff scores to inform diagnostic status [19,29,33]. Separate trauma-specific versions of symptom severity measures have also been developed (e.g., military vs. civilian [33]). In addition, more brief versions have been proposed to further reduce the time demands, but not the accuracy, of symptom severity questionnaires such as the Primary Care PTSD screen (PC-PTSD [25,34]).

## 3. Evidence-Based Treatments

Is PTSD primarily a biological or psychological phenomenon, and relatedly, are psychosocial or pharmacological treatments more appropriate? This question sets up a false dichotomy, as PTSD is rooted in both biological and psychological factors with regard to onset of symptoms, development of PTSD diagnosis, and maintenance of the disorder. Studies demonstrate that biological differences [36] and psychosocial differences [14,37] contribute to the risk for developing PTSD. Experimental research additionally provides evidence that both biological and psychological interventions delivered relatively soon after trauma exposure have the potential to mitigate or even prevent (in the case of psychotherapy for Acute Stress Disorder) the development of PTSD [38,39]. Furthermore, across several controlled clinical trials, both pharmacological [40] and psychological [41] interventions have been shown to significantly reduce PTSD symptoms. Altogether, the extant literature provides a strong case that PTSD is rooted in both biological and psychological underpinnings. The more pressing question, then, is which intervention pathway provides the most potent and persistent symptom reduction, and for which patients? The following section reviews evidence-based psychological and pharmacological treatments. 

### 3.1. Psychosocial Interventions for PTSD

**Exposure-Based Interventions.** Exposure-based interventions are the most empirically supported treatment modalities for PTSD [41,42]. The early roots of exposure-based therapies rest in the development of behaviorism in the 1920s, when Pavlov [42] demonstrated that fear could be both conditioned and extinguished through learning experiences. For example, repeatedly pairing the presentation of a tone with an uncomfortable shock eventually led to an automatic fear response to the tone (even in the absence of a shock). Furthermore, repeatedly playing the same feared tone without the shock eventually reduced (or extinguished) the fear response to the tone. Exposure-based behavioral therapies for PTSD are rooted in these same straightforward principles. The therapist helps the patient to systematically approach, instead of avoid, safe but feared stimuli (e.g., the memory of the trauma or situations that remind the patient of the traumatic event) in the absence of the feared consequences (such as bodily harm or unending anxiety), until the feared consequences are disconfirmed and the automatic fear response to trauma-related stimuli subsides. Though this basic principle is common to all exposure-based therapies across anxiety disorders, the necessity of defining the therapy provided in the context of clinical trials led to the development of specific, session-by-session exposure therapy protocols for the treatment for PTSD. 

One of the most commonly investigated and empirically-supported exposure-based protocols for PTSD is Prolonged Exposure therapy (PE; [41,43]). PE is an 8-to-15-session protocol, typically provided in weekly or bi-weekly, 60-to-90 minute sessions [43,44]. The majority of patients who complete PE evidence significant and reliable reductions in PTSD symptoms [45]. In the beginning of PE, patients are taught a brief relaxation breathing exercise, and they receive psycho-education about PTSD symptoms and factors that contribute to the maintenance of PTSD (e.g., avoidance of the memory and related reminders). Over the next several sessions, the patient revisits and describes the trauma memory aloud for a prolonged time (e.g., 30–45 min) in order to extinguish the fear response associated with the memory. This is called imaginal exposure. In addition, the patient is taught to approach safe, trauma-related situations that have been avoided because they remind the patient of the trauma. This is called in vivo exposure. As “homework” between sessions, patients listen to recordings of the therapy sessions and practice the in vivo exposures. 

In randomized clinical trials, dropout rates from PE have ranged from 10%–38% [44]. Notably, researchers have found that dropout can be higher in community settings ([46,47] although dropout rates do not differ between exposure and non-exposure therapies for PTSD [47]. A meta-analysis pooled across 13 studies found large effect sizes of PE relative to control groups at post-treatment, and medium to large effects at follow up time points [41]. Evidence also suggests that PE can produce further symptom reduction among patients with only partial response to pharmacotherapy [48]. 

### 3.2. Cognitive-Based Therapies 

Over time, the field of psychotherapy has expanded to include cognitive-based treatment techniques in addition to exposure-based techniques. Though PE is categorized as a cognitive-behavioral therapy, and its exposure-based protocol does produce changes in negative thinking patterns associated with PTSD [49], the intervention strategies themselves are primarily behavioral rather than cognitive. Cognitive Processing Therapy (CPT; [50,51]) on the other hand, relies more heavily on interventions that directly target maladaptive thinking patterns. CPT, alongside other cognitive-based therapies for PTSD, emphasizes the role that maladaptive or inaccurate interpretation of a situation plays in maintaining disorders such as PTSD, and intervenes directly with the thoughts rather than the resulting behaviors.

Similar to PE, the initial sessions of CPT include psycho-education about PTSD symptomatology and the role of avoidance in maintaining PTSD, but CPT provides much stronger emphasis on the role of maladaptive thinking patterns in maintaining PTSD symptoms. Early in therapy, the patient writes a statement of impact that the traumatic event had on their life, specifically including details about how the trauma affected the patient’s beliefs about self, others, and the world. This is read aloud and discussed with the therapist. The therapist begins to gently question any potential maladaptive thinking patterns, thereby helping the patient discover over-generalized or unhelpful automatic thoughts. Over time, the therapist works with the patient to develop strategies for generating more useful or accurate thinking patterns. In the standard CPT protocol, the patient additionally writes one to two detailed accounts of the trauma and reads this account aloud in session. The last several sessions focus on specific areas of one’s life that are likely affected by maladaptive trauma-related thought patterns, including the areas of safety, trust, power/control, esteem, and intimacy. At the end of treatment, the patient re-writes the impact statement, which is used to evaluate treatment gains. 

The 12-session CPT protocol can be disseminated effectively in either a group or individual format [51]. Elements of CPT have also been disseminated effectively in a dyadic format in the context of Cognitive Behavioral Conjoint Therapy (CBCT) for PTSD [52]. Dropout rates are approximately 20% in CPT [53] and no different than other active psychotherapies for PTSD [54,55]. Numerous trials have found CPT to be superior to a wait-list control group [53,55,56] and one study has demonstrated its equivalence to PE [55]. One dismantling study suggests that CPT may be equally efficacious with and without the written account of the trauma [57]. Subsequent analyses of this data qualified these results, demonstrating that those with higher levels of dissociation (especially depersonalization) responded best to the full protocol, and those with lower dissociation responded more rapidly to CPT without the trauma account [58]. Of note, other cognitive therapy protocols [59,60,61,62] or combined exposure and cognitive therapy protocols [63,64] have shown promising results [41]. 

### 3.3. Eye Movement Desensitization and Reprocessing. 

Eye movement desensitization and reprocessing (EMDR) therapy had also received empirical support for the treatment of PTSD [65,66]. The model used to explain PTSD in EMDR is similar to cognitive-behavioral therapies in that PTSD is viewed as a result of insufficient processing of the traumatic memory. EMDR hypothesizes that the trauma memory, if not fully processed, is stored in its initial state, preserving any misperceptions or distorted thinking patterns that occurred at the time of the trauma. 

At the outset of EMDR, patients are trained in strategies for managing negative emotions. The length of this treatment phase varies in accordance with the patient’s skill level in this area. To prepare for “reprocessing,” patients generate a list of traumatic (or other emotionally significant) experiences, along with distorted beliefs related to the experience (e.g., “I am a failure”) and desired beliefs (e.g., “I can handle tough situations”). During the reprocessing phase, the therapist asks the patient to bring to mind a vivid visual representation of the traumatic memory, along with the distorted belief (i.e., cognitive exposure), and to focus on the physical sensations related to the traumatic memory (i.e., interoceptive/visceral exposure). The patient is then instructed to engage in bilateral/saccadic eye movements, following the clinician’s finger from left to right for several repetitions. The patient visualizes the memory while continuing to engage in the bilateral stimulation. The patient is asked what experiences emerge next (e.g., thoughts, images, emotions, or sensations), and the cycle is repeated. The patient later practices thinking the desired thought (e.g., “I can handle tough situations”) with the visual image of the trauma brought to mind. 

The bilateral eye movements in EMDR are somewhat controversial. In support of the use of this strategy, van den Hout and colleagues [67] found that bilateral eye movements during autobiographical memory recall reduce vividness and emotions attached to the memory (though their research was conducted in healthy controls rather than in PTSD patients). Developers of EMDR hypothesize that bilateral eye movements therefore reduce distress attached to the trauma memory, thereby reducing avoidance, and allowing for increased attention to more adaptive thinking patterns that are then attached to the traumatic memory [65]. A recent meta-analysis further supports that bilateral stimulation (eye movements are not the only potential form of bilateral stimulation used in EMDR) impacts memory in ways that might facilitate PTSD treatment [68]. Other researchers have hypothesized, however, that the exposure-based components of EMDR are all that is required, and a review of dismantling studies has demonstrated that the EMDR protocol works just as well without the bilateral stimulation component [69]. Regardless of the validity of its theoretical underpinnings, EMDR has empirical support in that it consistently outperforms no-treatment controls and demonstrates similar outcomes to exposure- and cognitive-based psychotherapies for PTSD [41,70].

### 3.4. Relaxation-Based Psychotherapies 

Although less frequently studied and supported in the more recent literature, relaxation-based psychotherapies are another type of psychotherapy for PTSD. One of the most commonly investigated relaxation-based therapies for PTSD is Stress Inoculation Training (SIT; [71,72]). The treatment model is based on Lazarus & Folkman’s [73] conceptualization of stress resulting from perceived situational demands outweighing perceived resources to meet demands [74]. In this model, PTSD and other anxiety/stress disorders are maintained by ongoing perceptions of situational demands outweighing the available coping resources. The primary goal in SIT is to increase the patient’s sense of mastery over their anxiety, and to “inoculate” patients against future episodes of pervasive anxiety and stress. Treatment therefore focuses primarily on skills training in a vast array of anxiety-management strategies such as breathing retraining, muscle relaxation, negative-thought stopping, and restructuring/challenging maladaptive cognitions. Relaxation skills are trained and practiced in sessions using techniques such as behavioral rehearsal and imagery, modeling, and role-play. As treatment progresses, anxiety management strategies are practiced in the context of increasingly challenging and anxiety-provoking situations, including during graduated in vivo/situational exposures. Mastering the use of anxiety management skills in stressful situations is viewed as producing “inoculation” against future problems. 

In clinical trials for PTSD treatment, SIT outperforms wait-list and supportive counseling [75,76]. However, evidence suggests that PE is superior to both SIT and SIT combined with exposure [75]. The reduced efficacy of PE+SIT in comparison to PE could be due to the reduced dosage of therapeutic exposure to feared situations in the combined treatment, or perhaps could be due to a “kitchen sink” effect (i.e., rapid training in too many strategies rather than focused mastery of one strategy). 

## 4. Evidence-Based Pharmacological Treatments

Although psychotherapeutic interventions are the first and most supported option for the treatment of PTSD, there are several evidence-based pharmacological treatments available. In contrast to psychological interventions, pharmacotherapies can be provided in most clinical settings and require much less time and effort on the part of the patient (e.g., fewer and shorter appointments, no homework between visits). The foundation of pharmacological treatments is supported by a growing literature for the association between PTSD and dysregulations in neurotransmitter and neuroendocrine systems [77,78,79,80]. For the purposes of this review, we have focused on the current medication options with the most evidence, and therefore omitted older (e.g., monoamine oxidase inhibitors) and less studied options (e.g., mood stabilizers).

### 4.1. Selective Serotonin Reuptake Inhibitors

Most of the current research on pharmacotherapy for PTSD is focused on the selective serotonin reuptake inhibitors (SSRIs) to treat PTSD [40]. SSRIs have a broad effect on PTSD symptoms, including improvements in re-experiencing, avoidance, numbing, and hyper-arousal symptoms, and related quality of life improvements associated with the symptom reductions [81]. In terms of specific SSRIs, both sertraline and paroxetine have received FDA support for PTSD treatment and were superior to placebo in multisite clinical trials [81,82,83,84,85]. Other agents, such as fluvoxamine and citalopram, also have received support for the treatment of PTSD [86,87]. Interestingly, paroxetine also has been shown to potentially address cognitive deficits associated with PTSD, in addition to the clinical symptoms [88]. Longer trials of SSRIs (36 weeks) have been associated with a higher percentage of treatment response compared to the standard 12-week trials [89]. Unfortunately, independent of the duration of the trial, the discontinuation of SSRIs is associated with the relapse of PTSD symptoms [81,83,90]. In contrast, symptoms typically remain stable or continue to improve after completion of evidence-based psychotherapy for PTSD [91]. 

### 4.2. Other Promising Agents

There are several other agents that have received support in the pharmacological treatment of PTSD. These agents tend to effect specific sets of symptoms within PTSD, and are often studied in conjunction with an SSRI [81]. Two common examples are trazadone, an antidepressant serotonergic agent with a sedating side effect, and prazosin, an antiadrenergic agent that has been studied in the treatment of sleep and nightmares in PTSD. Prazosin has received increased attention as of late after randomized clinical trials demonstrating its effectiveness for PTSD-related sleep disruptions and nightmares, as well as global functioning and PTSD symptoms [92]. While these medications have shown some promise in reducing PTSD symptoms, they are not FDA approved for the treatment of PTSD and further research on their efficacy for PTSD is needed. 

## 5. Summary and Future Directions

In summary, PTSD is a relatively common and highly debilitating psychiatric disorder affecting approximately 8% of the U.S. population [2]. Potent evidence-based psychosocial interventions are available, and several medications have FDA approval for the treatment of PTSD. While pharmacological treatments have shown some promise, more investigation and advancement in this area is needed. One of the most important concerns with the sole use of pharmacotherapy for PTSD treatment is the evidence that discontinuing treatment can be associated with relapse [81,83,90]. Although relapse is relatively infrequent after one responds to an evidence-based psychotherapy for PTSD [91], a proportion of patients either drop out of therapy prematurely or do not respond to therapy [46,47,54]. It is therefore critical to continue to investigate new strategies to improve upon the available treatments for PTSD. 

One novel line of research has investigated the potential to enhance mechanisms of learning during cognitive behavioral therapies (such as those used for PTSD) by administering medications that could facilitate fear extinction, for example, *d*-cycloserine, yohimbine, methylene blue, MDMA, and oxytocin [93,94]. However, pharmacological augmentation of learning mechanisms is still in its infancy and will require much further exploration before these strategies can be recommended as standard treatment techniques for PTSD. Another line of cutting edge research involves priming the trauma memory through a reminder, and then preventing reconsolidation of the primed memory through pharmacological blockade [95]. Although some evidence suggests that this technique reduces emotional reactivity to the trauma memory [95], findings in this newer area of research are very preliminary and somewhat conflicted [39]. Innovative treatments outside the realms of psychotherapy and pharmacotherapy, such as neuronal feedback and brain stimulation techniques [96], are also being explored and may help reduce PTSD symptoms, particularly in treatment-resistant patients. 

## Figures and Tables

**Table 1:** Commonly used screening and self-report measures for trauma exposure and PTSD symptom severity.

Exposure to Potentially Traumatic Events		
Scale Name	Length	Reference
Brief Trauma Questionnaire	10 items	Schnurr et al., 2002 [23]
Trauma Assessment for Adults—Self Report	17 items	Gray, Elhai, Owen, & Monroe, 2009 [21]
Life Events Checklist for DSM-5 (standard version)	17 items	Gray, Litz, Hsu, & Lombardo, 2004 [24]
Traumatic Life Events Questionnaire	23 items	Kubany et al., 2000 [22]
**Brief Screening for Probable PTSD**		
Primary Care PTSD Screen	4 items	Prins & Ouimette, 2004 [25]
Short Form of the PTSD Checklist-Civilian Version	6 items	Lang & Stein, 2005 [26]
Short Post-Traumatic Stress Disorder Rating Interview	8 items	Connor & Davidson, 2001 [27]
Trauma Screening Questionnaire	10 items	Brewin et al., 2002 [35]
**Symptom Severity Self-Report Questionnaires**		
PTSD Checklist for DSM-5	20 items	Weathers et al., 2013 [28]
Posttraumatic Diagnostic Scale for DSM-5	24 items	Foa et al., 2015 [29]
